# Hepatoprotective effects of *Cassia* semen ethanol extract on non-alcoholic fatty liver disease in experimental rat

**DOI:** 10.1080/13880209.2019.1568509

**Published:** 2019-02-13

**Authors:** Yuanyuan Meng, Yong Liu, Ningning Fang, Yongmin Guo

**Affiliations:** Department of Anesthesiology, Qilu Hospital of Shandong University, Shandong, Jinan, People's Republic of China

**Keywords:** High-fat diet, antioxidant, low density lipoprotein receptor, TNF-α, IL-6, IL-8

## Abstract

**Context:***Cassia* semen (Cs), a seed of *Cassia obtusifolia* L. (Leguminosae), is a popular functional beverage. Previous studies reported that Cs displayed antioxidant, antifungal and strong liver protective effects.

**Objective:** This study evaluates the hepatoprotective effects of Cs on non-alcoholic fatty liver disease (NAFLD).

**Materials and methods:** Seventy-two male Wistar rats raised with high-fat diet (HFD) were randomly allotted into model, metformin (0.2 g/kg) and Cs (0.5, 1, and 2 g/kg)-treated groups. Another 12 rats were raised with normal feed as control group; all the rats were orally administrated with drugs and vehicle for 6 weeks. Alanine transferase (ALT), aspartate transaminase (AST), triglycerides (TG), total cholesterol (TC), malondialdehyde (MDA), superoxide dismutase (SOD), glutathione (GSH), tumor necrosis factor (TNF)-α, interleukin (IL)-6, IL-8 and low density lipoprotein receptor (LDL-R) mRNA levels were measured at the end of the experiment.

**Results:** Twelve weeks of HFD administration significantly increased the levels of AST, ALT, TG, TC, TNF-α, IL-6, IL-8 and MDA, decreased SOD (199.42 vs. 137.70 U/mg protein) and GSH (9.76 vs. 4.55 mg/g protein) contents, compared to control group. Cs administration group significantly decreased the elevated biomarkers with the ED_50_ = 1.2 g/kg for NAFLD rats. Cs treatment also prevents the decreased expression of LDL-R mRNA, and improved the histopathological changes compared to model group.

**Conclusions:** The hepatoprotective effect of Cs on NAFLD may possibly be due to its antioxidant effect. Cs may become a potent hepatoprotective agent in clinical therapy in the future.

## Introduction

*Cassia obtusifolia* L. (Leguminosae) is an annual herb that grows in tropical countries in Asia with a strong vitality. *Cassiae* semen (Cs), the seed of *Cassia obtusifolia*, is widely cultivated in China and usually consumed as a roasted tea by Chinese people (Ju et al. 2010). Due to the bioactive composition of Cs, it is very popular as a functional beverage. Cs mediates many pharmacological effects, for example, some research reported that the functional beverage made by Cs could reduce the levels of fat and cholesterol in serum, alleviate obesity, and also have an insulin resistance effect through up-regulating the AMP-dependent protein kinase (Fu et al. 2014; Kim et al. 2014). In traditional Chinese medicine (TCM), Cs has been usually used to treat dizziness and headache, to benefit eyes, and to nourish the liver. Kim et al. (2007, 2014) and Ip et al. (2016) reported that Cs could improve learning and memory impairment in mice, induced by scopolamine and transient cerebral hypoperfusion through mediation of the inhibition of acetylcholinesterase activity. Chen et al. (2017) reported that Cs also could alleviate acute lung injury in a rat model. Liang et al. (2009) studied the protective effect of ethyl acetate extract of Cs on experimental rats, which can significantly reduce the content of serum and liver total cholesterol, 3-acylglycerol, low density lipoprotein cholesterol and serum ALT and AST in the liver tissue, and increase the amount of high-density lipoprotein cholesterol, and improve the pathological damage of the liver.

In addition, current research reported that the main bioactive components of Cs are anthraquinone compounds, such as obtusin, emodin, aloe-emodin, etc. (Cao et al. 2015; Jung et al. 2016; Dong et al. 2017), which could reverse the changes of cytochrome P450 enzyme activity, and ultrastructure in rats induced by CCl_4_, showing a strong liver preservation effect (Wang et al. 2018) through its antioxidant activities. Although several pharmacological activities of anthraquinones have been reported (He et al. 2014; Kwon et al. 2018; Paudel et al. 2018), effects of these major bioactive constituents or the extracts of Cs on liver injury have not been investigated in the non-alcoholic fatty liver disease.

Non-alcoholic fatty liver disease (NAFLD) is one type of fatty liver, due to the fat depositing in liver for other than excessive alcohol use. NAFLD is considered to cover a wide spectrum of liver diseases such as hepatocellular carcinoma (HCC), simple steatosis, hepatic fibrosis (HF), hepatic cirrhosis, and non-alcoholic steatohepatitis (NASH) (Chalasani et al. 2012; Wilkins et al. 2013). Recently, approximately 20% of people worldwide and 25% of people in Western and Europe countries have NAFLD; it has become one of the most dominant causes for chronic hepatic disease in both adults and children (Ok et al. 2018). Among the patients with NAFLD, about 90% of patients have closely related with hypertension, dyslipidemia, obesity, elevated triglyceride levels, metabolic syndrome, insulin resistance, type 2 diabetes mellitus or cardiovascular disease, etc., it is increased especially in the older people (Schuppan and Schattenberg 2013). At present, the ‘two-hit’ theory has been widely accepted as a popular mechanism of NAFLD (Day and James 1998), which suggests that the lipid metabolism disorder triggered by insulin resistance is an important pathogenesis of NAFLD. However, there still is no therapeutic agent available and tested in clinical phase III trials, and there is no specific therapy can be firmly recommended to the patients with NAFLD (Chalasani et al. 2012; Nascimbeni et al. 2013).

Therefore, based on the above background, the present study is intended to investigate the potent efficient agents for NAFLD. Herein, to establish the NAFLD model in rats, we used the high-fat diet to administrate rats for 12 weeks, through detection the levels of liver functional enzyme (ALT, AST, TG and TC), antioxidant (SOD, GSH), peroxide products (MDA), the inflammation factors of TNF-α, IL-6, IL-8, and LDL-R mRNA levels to evaluated the possible beneficial effects and the possible action mechanism of Cs ethanol extract on NAFLD in rats.

## Materials and methods

### Chemicals

Metformin (MET) was purchased from Selleck Chemicals (USA). Alanine aminotransferase (ALT), aspartate aminotransferase (AST), triglycerides (TG), total cholesterol (TC), malondialdehyde (MDA), superoxide dismutase (SOD) and glutathione (GSH) kits were obtained from Nanjing Jiancheng Bioengineering Institute (China). The ELISA kits of tumor necrosis factor (TNF)-α, interleukin (IL)-6 and IL-8 were purchased from R&D Company (USA).

All the other chemicals and solvents used in this study were of highest purity and were purchased from Sigma-Aldrich Chemicals (USA).

### High fat diet preparations

The high fat diet (HFD) contained 88% basic feed (Beijing Botai Hongda Biotechnology Co. Ltd), 10% lard (Yichang Shuanghui Food LLC) and 2% cholesterol (CAS: 57-88-5). To prepare, first, weigh a certain amount of the basic feed, lard, and cholesterol. Then the lard is put in a pot and heated at 40 °C to melt the lard; next the weighted cholesterol is added and mixed well; finally the basic feed is added and mixed well, then the entire mixture is cooled to room temperature, wind dried, and stored in a well ventilated place.

### Experimental animals

This study was conducted using male Wistar rats with body weight 140 ± 10 g, which were obtained from Experimental Animal Center of Shandong University (China). Six rats were kept in one polyacrylic cage, and all the rats were quarantined for 1 week before the experiments. All animals were housed under standard controlled conditions with temperature maintained at 24 ± 2 °C, humidity: 50% ± 5% and 12 h light/dark cycle. The rats were provided free access to food (standard commercial rat chow) and water, and received human care according to National Institutes of Health Guidelines of United States (National Research Council of United States, 1996) and related ethical regulations of Shandong University. The animal experiment protocol was approved by animal ethics committee of Shandong University (Protocol number: 2016Guo-09). Animals were fasted for 12 h before tissue obtained.

### Preparation of ethanol extract of *Cassia* semen

*Cassia* semen was purchased from Shunchang herb store (Shanghai, China) on 8th November 2016, authenticated by Zhengchen Zhang at the Zhejiang Institute of Drug Control (Hangzhou, China), and the specimen stored at the Department of Anesthesiology, Qilu Hospital of Shandong University (specimen no: 20170106). The air-dried Cs were ground to powder for extraction. Powdered Cs (1 kg) was macerated with 8 L 70% ethanol for 1 h at room temperature, then reflux at the temperature of 60 °C with water bath. The supernatant was then collected and filtered through Whatman No. 1 filter paper in a Buchner funnel under vacuum. The filtrate was concentrated by evaporation with a vacuum rotary evaporator at 45 °C. The extract was freeze-dried at reduced pressure and stored at 4 °C for the experimentation. Freeze-dried powdered Cs (1 g) is equivalent to 8.35 g of the raw materials of Cs. Further, the vacuum-packed freeze-dried powdered Cs could be stored in stable condition in a cool, dry place away from sunlight for 24 months.

### Experimental design

After acclimatization for one week, 60 rats were used to establish the NAFLD model with a HFD for 12 weeks and they were randomly alloted into five groups (*n* = 12): model group, metformin group (0.2 g/kg BW orally administration once in a day for six weeks) and Cs treated groups (0.5, 1, and 2 g/kg BW orally administration once in a day for six weeks, respectively), while another 12 male rats were raised with normal feed, and regarded as the normal group. On week 12, all the rats were sacrificed by cervical dislocation at the end of the experiment. Blood samples were harvested for serum (3000 rpm, 10 min, and 4 °C) biochemical markers assay. The fresh liver obtained and weighed to calculate liver coefficient (Liver coefficient %=liver weight/body weight × 100). The right liver lobe was fixed in 10% formalin to prepare paraffin sections and the rest was stored at −80 °C until assays.

### Biochemical assays

Serum biochemical marker ALT, AST, TG and TC were detected using commercial kits according to the manufacturer’s instructions by a multifunctional biochemistry analyzer Olympus AU600 (Olympus, Japan). The observation absorbance of ALT and AST were read at 505 nm and the enzyme activity was calculated as U/L. The observation absorbance of TG and TC was read at 510 nm and the content was calculated as mmol/L. The serum levels of TNF-α, IL-6 and IL-8 were analyzed according to the ELISA kit protocol from R&D, which obtained the absorbance at 450 nm. The values of TNF-α, IL-6 and IL-8 were calculated as ng/mL, pg/mL and ng/mL, respectively (Crowther 2002).

Liver homogenate (10%, w/v) was prepared by homogenizing the liver tissue in 150 mmol/L Tris-HCl buffered saline (pH 7.2) with a polytron homogenizer. The level of MDA in liver tissues was measured at 532 nm with a spectrophotometer following the kit protocol from Jiancheng Biological Engineering Institute (Nanjing, China). The data are expressed as nmol/mg protein of liver tissue.

The activity of SOD and GSH were determined by commercial kits from Jiancheng Biological Engineering Institute (Nanjing, China) following the protocol provided by manufacture. The observation absorbance of SOD reaction was read at 550 nm and the data are expressed as U/mg protein, while GSH reaction was read at 420 nm and the enzyme activity was calculated as mg/g protein.

### Histopathological study

After dehydration, the right liver lobes fixed in 10% formalin buffer, then embedded in paraffin and cut into 5 µm thickness according to the routine procedure. The serial sections were stained with hematoxylin and eosin (H&E) for routine histopathological examination, and examined under a light microscope (Olympus BX-50 Microscope, Leica Microsystems, Germany) at 200 × magnification for the degree of hepatic steatosis and photographed. In addition, the pathological stages of hepatic steatosis were determined according to Metavir scoring system (Zou et al. 2015), F0 stage: normal liver tissue, no fibrosis; F1 stage: fibrosis extended to some portal areas; F2 stage: fibrosis extended to most portal areas; F3 stage: fibrosis extended to most portal areas, and portal fibrosis can be bridged opportunely; F4 stage: fibrosis extends to most portal areas, and there is characteristic bridging between portal fibrosis and central lobular fibrosis; F5 stage: there is characteristic bridging between portal fibrosis and central portal fibrosis, and there is opportunistic nodule formation.

### Quantitative RT PCR analysis

Total RNA samples of liver tissue were extracted by TRI pure reagent following the manufacturer’s protocols in each experimental group. Use UV spectrophotometer to measure the absorption values (A) in 260 and 280 nm to calculate the purity and concentrations. cDNA was obtained from the reverse transcription of total RNA (2 μg) from each samples, according to PrimeScript®RT reagent kit with a TC-512 PCR system (TECHNE, UK). PCR reaction system (25 μL) as follows: 5 μL 10 × buffer, 1 μL dNTP, 5 μL Taq DNA Polymerase, 0.25 μL LDL-R forward primer, 0.25 μL LDL-R reverse primer, 0.25 μL β-actin forward primer, 0.25 μL β-actin reverse primer, 1 μL cDNA template, 17 μL DEPC water. The β-actin gene was amplified separately as an internal control to normalize for gene expression in the samples. The primer sequences were as follows: β-actin (304 bp): forward 5′-AATGAGCGGTTCCGATGC-3′, reverse 5′-CGAAGGTGGACAGTGAGGC-3′, LDL-R (221 bp): forward 5′-CAAGGACCTCAAGATTGGCTATG-3′, reverse 5′-AGAGCAGAAACCCTAT GGAACC-3′. The reaction conditions were as follows: pre-denature at 94 °C for 4 min, denature at 94 °C for 20 s, annealing at 58 °C, 20 s for LDL-R and 54 °C, 20 s for β-actin, extension at 72 °C for 40 s and 72 °C, 7 min for the last cycle, totally 32 cycles, then stored at 4 °C. The changes in the model group, MET-treated group, Cs-treated groups and the control group was calculated as the absorption values of LDL-R/β-actin (Livak and Schmittgen 2001).

### Statistical analysis

Data were presented as means ± SD. One-way ANOVA was carried out, and all the statistical comparisons among the groups were performed with Dunett’s *t*-test using a Statistical Package for the Social Sciences 17.0 (SPSS version 17.0).

## Results

### Effects of Cs on body weight and liver coefficient

**Figure 1.**
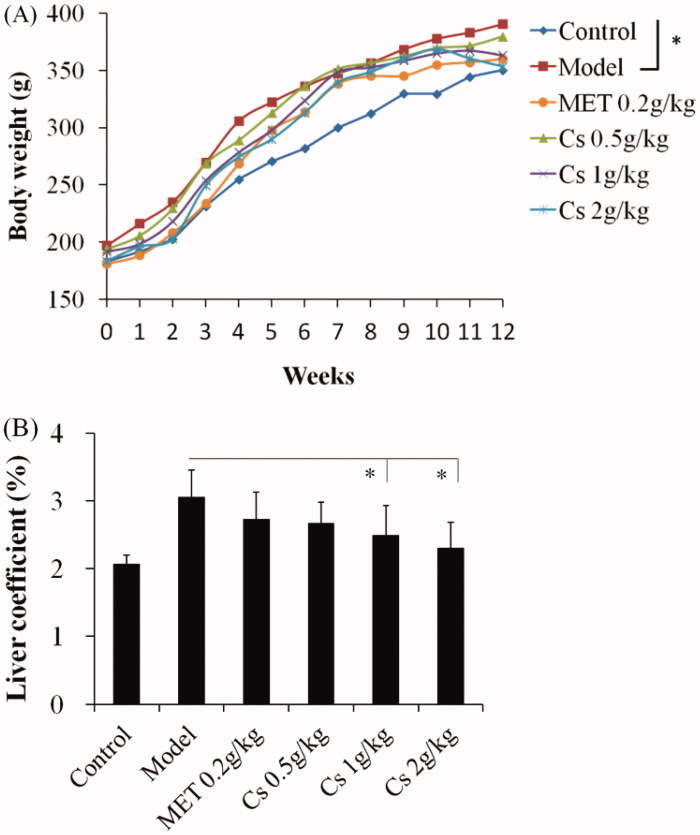
Effects of *Cassia* semen ethanol extract on body weight (A) and liver coefficient (B). *p < 0.05 *vs.* model group. Cs: *Cassia* semen, MET: Metformin.

At the end of the experiment, the body weight of rats in model group remarkably increased compared that of the control group (*p* < 0.05, Figure 1(A)). While the gain of the body weight in Cs-treated groups was lower than that of model group in some extent (Figure 1(A)), which may reflect that Cs treatment could inhibit the occurrence of obesity in long term HFD administrated rats. In addition, the liver coefficient was also reduced markedly in Cs-treated rats, especially the higher Cs dosage group, compared to model group (*p* < 0.05, Figure 1(B)).

### Effect of Cs on serum ALT, AST and blood lipid levels

As shown in Table 1, serum levels of AST and ALT were significantly increased in model group compared with the control group (*p* < 0.01). However, the serum levels of AST and ALT were significantly reduced with a dose-dependent manner in Cs treated groups (0.5, 1, and 2 g/kg), compared with the model group (*p* < 0.05, *p* < 0.01, Table 1).

**Table 1.** Effect of *Cassia* semen ethanol extracts on serum liver function markers and blood lipid levels.

Group	ALT ( IU/L)	AST ( IU/L)	TG ( mmol/L)	TC (mmol/L)
Control	86.72 ± 9.60	160.65 ± 10.23	0.70 ± 0.05	1.81 ± 0.46
Model	261.25 ± 33.55^##^	362.17 ± 38.23^##^	1.36 ± 0.32^##^	2.64 ± 0.61^##^
MET 0.2 g/kg	176.33 ± 22.06**	268.53 ± 9.18*	0.75 ± 0.06*	1.50 ± 0.31*
Cs 0.5 g/kg	230.90 ± 27.84	305.91 ± 13.65*	1.08 ± 0.13	2.01 ± 0.24
Cs 1 g/kg	203.33 ± 20.06*	288.53 ± 9.18*	0.85 ± 0.31*	1.70 ± 0.28*
Cs 2 g/kg	180.77 ± 13.52**	238.44 ± 9.79**	0.65 ± 0.13**	1.33 ± 0.35**

Data are expressed as the mean ± SD (*n* = 12) in each group. ^##^*p* < 0.01 *vs* control group; *p < 0.05, **p < 0.01 *vs* model group. Cs: *Cassia* semen, MET: Metformin; ALT: Alanine transferase; AST: Aspartate transaminase; TG: Triglycerides; TC: Total cholesterol.

In addition, HFD induced NAFLD provoked a significant increase in the levels of TC and TG compared with control group (*p* < 0.01, Table 1), which reflected that the successful NAFLD model has been established in rats. From Table 1, we could see that, after Cs treatment, the levels of both TC and TG in blood were markedly decreased with dose-dependent manners compared to NAFLD model group (*p* < 0.05, *p* < 0.01, Table 1), which indicated that Cs had an obvious lipid-lowering effects in the process of NAFLD.

### Effects of Cs on liver tissue SOD, GSH and MDA levels

Table 2 showed a significant difference in the levels of antioxidant between model group and the control group. In the model group, the levels of SOD and GSH were largely decreased compared with that of the control group (*p* < 0.01, Table 2). However, after 6 weeks Cs treatment (0.5, 1, and 2 g/kg), the levels of SOD and GSH were all significantly increased with a dose-dependent manner, compared with the model group (*p* < 0.05, *p* < 0.01, Table 2). While like the previous study, the MET treated group also increased the levels of the antioxidant SOD and GSH, compared to the model group (*p* < 0.05, Table 2).

**Table 2.** Effect of *Cassia* semen ethanol extracts on liver antioxidant enzyme-specific activities, antioxidant and lipid peroxidation levels.

Group	SOD (U/mgprot)	GSH (mg/gprot)	MDA (nmol/mgprot)
Control	199.42 ± 19.23	9.76 ± 1.32	4.19 ± 1.13
Model	137.70 ± 7.89^##^	4.55 ± 0.91^##^	9.58 ± 3.02^##^
MET 0.2 g/kg	177.95 ± 9.51*	6.68 ± 1.26*	5.04 ± 1.41*
Cs 0.5 g/kg	140.95 ± 7.18*	5.88 ± 1.06*	5.24 ± 1.46**
Cs 1 g/kg	159.50 ± 11.13*	6.79 ± 0.93*	4.13 ± 1.13**
Cs 2 g/kg	180.25 ± 10.79**	7.79 ± 1.48**	3.45 ± 1.39**

Data are expressed as the mean ± SD (*n* = 12) in each group. ^##^*p* < 0.01 *vs* control group; *p < 0.05, **p < 0.01 *vs* model group. Cs: *Cassia* semen, MET: Metformin; SOD: Superoxide dismutase; GSH: Glutathione; MDA: Malondialdehyde.

In addition, HFD administration model group caused a significant increase in the concentration of MDA when compared with the control group (9.58 vs. 4.19 nmol/mg protein, *p* < 0.01, Table 2). However, treatment with 0.5, 1, and 2 g/kg of Cs markedly reduced the amount of MDA in the liver tissue, respectively, compared to the model group (*p* < 0.01, Table 2).

### *Effects of Cs on serum TNF-*α*, IL-6 and IL-8 levels*

As shown in Table 3, HFD administration model group showed a significant increase in the levels of TNF-α, IL-6 and IL-8, compared with the control group (*p* < 0.01, Table 3). In contrast, the rats treated with Cs (0.5, 1, and 2g/kg) showed remarkably decreased levels of TNF-α, IL-6 and IL-8, compare with the model group (*p* < 0.05, *p* < 0.01, Table 3).

**Table 3.** Effect of *Cassia* semen ethanol extracts on serum levels of TNF-*α*, IL-6 and IL-8.

Group	TNF-*α* (ng/mL)	IL-6 (pg/mL)	IL-8 (ng/mL)
Control	0.85 ± 0.30	61.33 ± 9.02	0.69 ± 0.11
Model	1.48 ± 0.51^##^	102.22 ± 36.19^##^	1.28 ± 0.42^##^
MET 0.2 g/kg	0.93 ± 0.29**	80.03 ± 8.44*	0.76 ± 0.14*
Cs 0.5 g/kg	1.52 ± 0.36	90.07 ± 10.55*	0.98 ± 0.23
Cs 1 g/kg	0.93 ± 0.21*	75.52 ± 6.66*	0.75 ± 0.11*
Cs 2 g/kg	0.86 ± 0.15**	63.41 ± 10.09**	0.68 ± 0.20**

Data are expressed as the mean ± SD (*n* = 12) in each group. ^##^*p* < 0.01 *vs* control group; *p < 0.05, **p < 0.01 *vs* model group. Cs: *Cassia* semen, MET: Metformin; TNF-*α*: tumor necrosis factor-*α*; IL-6: Interleukin-6; IL-8: Interleukin-8.

### Effects of Cs on histopathological changes in the liver tissue

**Figure 2.**
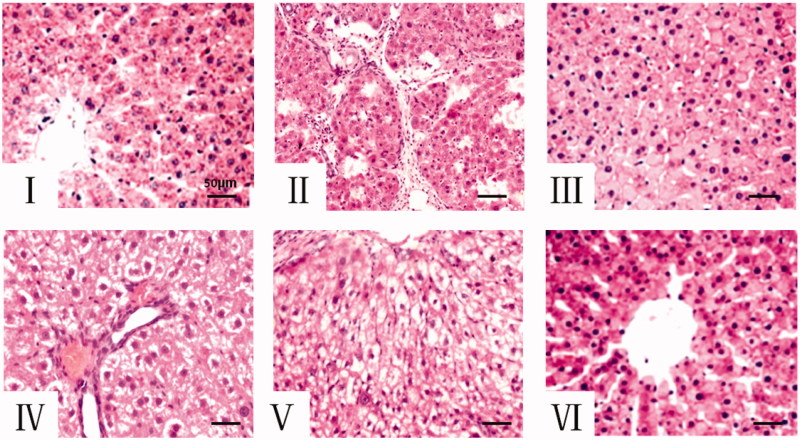
The histopathological examination by H&E (200 ×). (A): Control group, (B): Model group, (C): MET 0.2 g/kg group, (D): Cs 0.5 g/kg group, (E): Cs 1 g/kg group. (F): Cs 2 g/kg group. Cs: *Cassia* semen, MET: Metformin.

The liver pathology sections stained by H&E and the pathological grade assessed by METAVIR scoring system are shown in Figure 2 and Table 4. Under the photomicroscope, the liver sections showed a clear, normal liver lobular architecture, the single liver cells showed a well-preserved cytoplasm and well-defined nucleus in the control group (Figure 2(A)). In the model group (Figure 2(B)), full fat and widespread lipid vacuoles in lobule cells, infiltration of inflammatory cells and lipid degeneration could be observed in central of the lobules. While, in the group of Cs, the microvesicular fatty, inflammatory infiltration and lipid degeneration were remarkably alleviated, especially in the high dose of Cs (2 g/kg) group, compared to the model group (Figure 2(F)).

**Table 4.** Grading of hepatic fatty lesions and inflammation of each group.

Group	*n*	F0	F1	F2	F3	F4
Control	12	12	0	0	0	0
Model	12	0	0	0	1	11
MET 0.2 g/kg	12	0	6	6	0	0
Cs 0.5 g/kg	12	0	6	3	3	0
Cs 1 g/kg	12	0	9	3	0	0
Cs 2 g/kg	12	3	8	1	0	0

Cs: *Cassia* semen; MET: Metformin.

### Effects of Cs on the molecular expression of LDL-R mRNA

**Figure 3.**
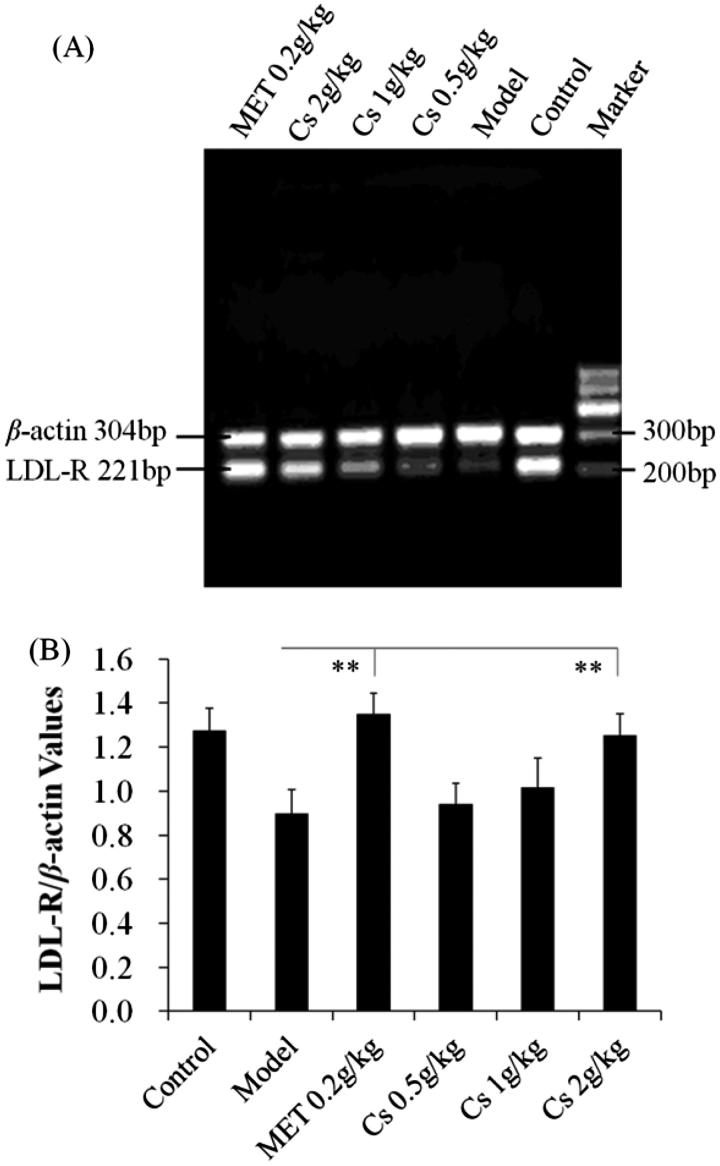
(A) Effect of *Cassia* semen ethanol extract on mRNA expression of LDL-R in liver tissue. (B) Data were expressed as mean ± SD of 12 rats in each group. **p < 0.01 *vs*. model group. Cs: *Cassia* semen, MET: Metformin.

In the present study, we detected the effects of Cs ethanol extract on the mRNA expression levels of LDL-R (Figure 3). It was obvious that the mRNA expression of LDL-R was dramatically down-regulated in the model group, compared to the control group (*p* < 0.01, Figure 3). However, the Cs-treated groups significantly increased the mRNA expression levels of LDL-R, especially the 2 g/kg Cs group, compared to the model group (*p* < 0.01, Figure 3). The quantitative analysis of the mRNA expression of LDL-R was calculated by the absorption values of LDL-R and β-actin by Image J software, which is expressed as the mean ± SD (*n* = 12).

## Discussion

Non-alcoholic fatty liver disease (NAFLD) is the most common form of chronic hepatic disease with manifestation of over-accumulation of fat instead of excessive alcohol intake in liver. It is associated with metabolic syndrome and the insulin resistance (IR) (Cohen et al. 2011; Magkos et al. 2011; De Souza Mesquita et al. 2018), it often developed a histological spectrum starting from simple steatosis to NASH with the characterization of hepatocellular damage, fibrogenesis and lobular necro-inflammation (Rath et al. 2016; Jin et al. 2018), which may evolve to hepatic cirrhosis and HCC (Liu et al. 2014; Andronescu et al. 2018). Recently, the animal models of NAFLD induced by HFD are considered as more close to human NAFLD in the aspect of pathophysiology, including obesity, IR and hepatic steatosis in mice or rats, although this model need a lengthy feeding period (Carabelli et al. 2011). According to the traditional Chinese medicine theory, the etiologic of NAFLD is related to the emotional disorders or poor diet with the key points of blood stasis and phlegm, and which will further affect the organs of liver, spleen, and kidney (Dong et al. 2012). Thus, in the current study, it is considered that promoting blood circulation to remove meridian obstruction, reducing phlegm, removing dampness and liver-kidney-tonifying are an effective method for NAFLD treatment. At present, there are no approved treatment drugs for NAFLD in the clinic, although tremendous effort have made in NAFLD prevention from clinicians and researchers. Therefore, it is very important and very valuable for development of a novel agent to delay or reverse the pathogenesis progression in NAFLD.

Based on the present study, it revealed that HFD induced NAFLD resulted in decreased in a decreased antioxidant enzymatic activity in liver such as SOD and GSH. Since these antioxidant enzymes catalyze the decomposition of ROS, the decrease of SOD and GSH activities might predispose the examined rat tissue to oxidative stress (Wang et al. 2015; Yu et al. 2017; Koca-Caliskan et al. 2018). The levels of these antioxidants might provide a clear indication on the extent of cytotoxic damage that occurs in liver tissue. In addition, MDA is an important lipid peroxidation index in many organ homogenate (Koca-Caliskan et al. 2018). The present study showed that the liver MDA content in model group was significantly increased compared to control group, which reflects that HFD administration increased the oxidative stress in rat. It is known that HFD-induced tissue damage by oxidative stress could be caused by two mechanisms: increased generation of ROS, and by causing direct depletion of antioxidant reserves (Yu et al. 2017; Cole et al. 2018). The components of Cs ethanol extract behave as powerful antioxidant and free radical scavenger, which can increase the activity of antioxidant enzyme (SOD and GSH) and decrease lipid peroxides MDA levels in liver of NAFLD rats, as observed in the study. Treatment with Cs extract at a dose of 0.5, 1, 2 g/kg BW significantly prevented the increased levels of lipid peroxidation (MDA) when rat were challenged with HFD, and also markedly increased the levels of SOD and GSH, which means that ethanol extract of Cs could decrease the liver damage induced by HFD through its antioxidant activity.

Liver damage displayed in NAFLD is well characterized by elevated levels of serum hepatic marker enzymes. In preclinical studies, the serum enzyme markers (such as AST and ALT) are regarded as a more specific and sensitive liver damage indicators, and also are recommended for the assessment of hepatocellular injury. While the liver injury from NAFLD will also lead to high levels of serum TG and TC, which are also considered as the key markers for liver damage assessment in NAFLD model. Generally, low levels of AST, ALT, TG and TC can be found in the blood, but when the liver is damaged, it will release AST, ALT, TG and TC into the blood stream, which results in a rise in AST, ALT, TG and TC levels (Wilkins et al. 2013; Yki-Jarvinen 2016; Lonardo et al. 2017; Tsai & Lee 2018). In the present study, we found that treatment with Cs ethanol extract could minimize the elevated serum levels of AST, ALT, TG and TC, which reflect that the protective effects of Cs for HFD induced NAFLD.

Furthermore, TNF-α is considered to be the major cytokine in the progression of simple fatty liver to NASH. In addition, TNF-α is a potent inhibitor of lipoprotein esterase. High concentrations of TNF-α can reduce lipolysis in peripheral tissues and promote the synthesis and aggregation of triglycerides in liver cells. The deposition of excess lipids, especially triglycerides, in the liver is a prerequisite for the formation and development of NAFLD. There was a significant positive correlation between serum TNF-α and elevated serum endotoxin levels (Amber et al. 2015). IL-6 can be produced by many kinds of cells, such as monocytes and macrophages, and is related to local inflammation of the liver. IL-6 participates in the process of inflammatory injury, and can inhibit lipolysis, inhibit lipoprotein esterase activity, and promote fatty liver formation (Koga et al. 2018). IL-8 can cause inflammatory cells such as activated neutrophils to accumulate in the liver and cause inflammation. The accumulation of lipids in hepatocytes and the elevation of endotoxin levels can also stimulate the release of TNF-α from Kupffer cells, which further promote the production of IL-8, and participate in the inflammatory response of hepatocytes, leading to the injury of hepatocytes (Tong et al. 2018). In the present study, as the same with the previous study, HFD administration significantly increased the serum levels of TNF-α, IL-6 and IL-8, while Cs treated groups dramatically reduced the elevated levels of TNF-α, IL-6 and IL-8. This result implied that the mechanism of action of Cs for NAFLD may be related with the regulation of serum cytokines.

In addition, histopathological changes in liver from microscopy observation correlated with the examination of liver function (Rath et al. 2016; Koo et al. 2017). In the present study, histopathological view of liver sections in HFD induced NAFLD model group showed loss of the normal structure of hepatic cells, widespread lipid vacuoles, ballooning degeneration, infiltrating lymphocytes and fatty degeneration. While after Cs ethanol extract treatment, the liver damage was significantly decreased in the dose of 1 and 2 g/kg BW but not with 0.5 g/kg BW, which is consistent with the data of liver functional makers. These results suggested that the inhibition of the elevation of liver function markers, obvious lipid-lowering and liver damage may related in the protective effect of Cs against HFD-induced NAFLD.

In the study, we also detected the levels of LDL-R mRNA in liver tissue. Increasing evidence indicates that NAFLD may be in part caused by malfunction of low density lipoprotein (LDL) secretion (Yu et al. 2017). Liver expression of the LDL-R is a major factor in regulation of plasma levels of LDL cholesterol (LDL-C) (Ivaturi et al. 2014). Human patients with loss-of-function LDL-R could develop to familial hypercholesterolemia (Usifo et al. 2012), and the homozygous familial hypercholesterolemia patients could develop to advanced atherosclerotic lesions at an early age (López et al. 2018; Tada et al. 2018). Thus, LDL-R could cause the metabolism outlet of all kinds of lipoprotein, and regulate the content of plasma lipoprotein (Go 2015; Toldo et al. 2017). In the present study, the levels of LDL-R mRNA in HFD-induce NAFLD model group was significantly decreased compared with the control group. However, Cs treatment groups (0.5, 1, and 2 g/kg) markedly up-regulated the levels of LDL-R mRNA in a dose-dependent manner, which further confirmed the protective effects of Cs ethanol extract.

In summary, the present study indicated that HFD-induced NAFLD might be related to oxidative damage and lipid disorders. Treatment with Cs extract lessened the effects of HFD-induced NAFLD, possibly by increasing the activity of antioxidant enzyme, inhibiting the MDA in liver and up-regulated the expression of LDL-R to regulate the lipid metabolism process. Further detailed studies of these promising protective effects of Cs extract against HFD-induced NAFLD may have a meaningful impact on developing feasible therapy strategies to patients with HFD-induced NAFLD.

## Conclusions

Taken together, oral administration of Cs ethanol extract for 6 weeks significantly reduced the serum levels of AST, ALT, TC and TG, decreased the serum cytokine levels of TNF-α, IL-6 and IL-8, increased the activity of the antioxidant SOD and GSH, decreased the amount of lipid peroxidation products in liver tissue, and further up-regulated the expression of LDL-R mRNA, improved the histopathological changes with a dose-dependent manner. It could be concluded that the Cs ethanol extract may exert a potential preventive effect against HFD induced NAFLD in rats, which possibly through its antioxidant mechanisms, remove free radicals, reduce the lipid peroxidation products, regulate the cellular cytokines, and up-regulated the expression of LDL-R.
